# Comparative study of two laboratory techniques for the detection of HLA-B27 in patients with axial spondyloarthritis: a cross-sectional analysis

**DOI:** 10.1186/s42358-024-00383-x

**Published:** 2024-05-23

**Authors:** Ricardo dos Santos Angeli, André Lucas Ribeiro, Charles Lubianca Kohem, Ricardo Machado Xavier, Odirlei André Monticielo

**Affiliations:** 1 Hospital de Clínicas de Porto Alegre, Faculdade de Medicina da Universidade Federal do Rio Grande do Sul, Porto Alegre, Brazil; 2 Serviço de Reumatologia, Hospital de Clínicas de Porto Alegre - HCPA, Faculdade de Medicina da Universidade Federal do Rio Grande do Sul, Rua Ramiro Barcelos, 2350, Porto Alegre, Rio Grande do Sul 90035-903 Brazil

**Keywords:** Ankylosing spondylitis, Axial spondyloarthritis, HLA-B27, Flow cytometry, PCR, Health economics

## Abstract

**Background:**

The diagnostic and prognostic relevance of Human Leukocyte Antigen B-27 (HLA-B27) in Axial Spondyloarthritis (AxSpA) is undeniable, with 70% of Ankylosing Spondylitis (AS) patients carrying the B27 gene, contrasted with a mere 4.35% in the general population. Flow cytometry (FC) and Polymerase Chain Reaction (PCR) have emerged as the predominant techniques for routine HLA-B27 typing. While various studies have compared these methods, none have catered to the unique characteristics of the Brazilian demographic. Therefore, this research aims to compare FC and PCR in a Brazilian cohort diagnosed with AxSpA.

**Methods:**

An analytical cross-sectional study was undertaken involving 62 AxSpA outpatients from a Brazilian University Hospital. Both FC and PCR-SSP assays were utilized to ascertain HLA-B27 typing. The outcomes (either confirming or refuting the allele’s presence) underwent rigorous scrutiny. Agreement between the methodologies was assessed using the kappa statistic. A *p*-value of < 0.05 was deemed statistically significant.

**Results:**

Of the participants, 90.3% (*n* = 56) were HLA-B27 positive according to FC, while 79% (*n* = 49) were identified as positive using the PCR method. FC exhibited a sensitivity rate of 98% paired with a specificity of 38.5%. The Positive Predictive Value for FC stood at 85.7%, and the Negative Predictive Value was 83.5%. Consequently, the overall accuracy of the FC method was gauged at 85.5%. A kappa coefficient of κ = 0.454 was derived.

**Conclusions:**

FC demonstrated noteworthy sensitivity and satisfactory accuracy in HLA-B27 detection, albeit with a reduced specificity when contrasted with PCR-SSP. Nevertheless, given its cost-effectiveness and streamlined operation relative to PCR, FC remains a pragmatic option for preliminary screening in clinical practice, especially in low-income regions. To optimize resource allocation, we advocate for a refined algorithm that initiates by assessing the relevance of HLA-B27 typing based on Choosing Wisely recommendations. It then leans on FC, and, if results are negative yet clinical suspicion persists, advances to PCR. This approach aims to balance diagnostic accuracy and financial prudence, particularly in regions contending with escalating medical costs.

## Introduction

Axial spondyloarthritis (AxSpA) encompasses a spectrum of chronic inflammatory disorders primarily targeting the axial skeleton, potentially resulting in structural ankylosis [1]. Besides the spine, entheses and peripheral joints are commonly involved, frequently leading to joint destruction and consequent physical disability [2]. The presence of radiographic changes in the sacroiliac joints delineates Ankylosing Spondylitis (AS), also known as radiographic axial spondyloarthritis (r-AxSpA), from its counterpart, non-radiographic Axial Spondyloarthritis (nr-AxSpA) [3–5].

AxSpA significantly impacts the quality of life of those affected, with the health impact being influenced by individual-level and country-level socioeconomic factors [6]. R-axSpA prevalence in Latin America falls between 0.2% and 0.8% [7], primarily affecting young adults in their twenties and thirties [8]. The advent of novel AxSpA therapeutic strategies underscores the necessity of prompt diagnosis and meticulous clinical profiling [9]. Diagnosing AxSpA in individuals with nonspecific chronic back pain involves an intricate process. This includes an assessment of patient demographics, back pain attributes, inflammatory markers like C-reactive protein, imaging of the axial skeleton, and importantly, the detection of Human Leukocyte Antigen-B27 (HLA-B27)—a dual diagnostic and prognostic instrument [10].

The relationship between HLA-B27 and AxSpA is well-established, yet its precise pathophysiological mechanisms remain incompletely understood [11]. Notably, the relative risk that HLA-B27 imparts in AS exceeds its influence in other conditions with genetic predispositions dictated by the MHC [12]. Globally, HLA-B27 seropositivity is observed in 90 to 95% of Caucasian AS patients [13]. In Brazil, a country with significant genetic diversity, the prevalence of HLA-B27 in AS is estimated to be between 65 and 70% [7, 14], a figure significantly higher than the 4.35% found in the general population [15]. Importantly, a positive HLA-B27 status correlates with a more severe disease phenotype and poorer functional status [16]. Due to its invaluable prognostic insights, the Brazilian Society of Rheumatology recommends HLA-B27 testing for patients with clinically suspected AxSpA for prognostic reasons [17].

Given the profound implication of HLA-B27 in the diagnosis and prognosis of AS, accurate detection becomes imperative. Traditional methods for HLA-B27 detection encompass serological tests and flow cytometry (FC). However, the specificity of these methods, at times, undergoes scrutiny, prompting medical professionals to gravitate towards genotyping techniques like polymerase chain reaction (PCR), which are more specific [18–20]. Nonetheless, FC remains widely used due to its cost-effectiveness, ease of use, prompt results, and high sensitivity. In contrast, PCR offers heightened specificity, albeit at a steeper cost and with increased procedural complexity. Hence, HLA-B27 detection by FC is still adopted in smaller diagnostic laboratories, especially in low-income regions like Latin America and India [21], but also in high-income regions like the US [22].

In light of the critical role HLA-B27 plays in AxSpA diagnosis and prognosis, ensuring its precise detection is of paramount importance. Although FC offers a blend of cost-effectiveness, simplicity, and sensitivity, it may be contrasted with the specificity promised by PCR, albeit at a higher cost and complexity. Yet, as healthcare decisions often pivot on the delicate balance of cost, accuracy, and resource availability, understanding the relative merits of these two diagnostic modalities is essential. With HLA-B27 prevalence differing across populations, this study in Brazil is key to gauging local predictive values. Our research, the first of its kind in the Brazilian population, contrasts FC and PCR for HLA-B27 detection, seeking the optimal mix of accuracy and feasibility. Beyond bridging a knowledge gap, our findings could steer clinical and policy directions, notably in settings with limited resources.

## Materials and methods

### Patients and study design

In this cross-sectional study conducted in 2015, we consecutively recruited adults over the age of 18 diagnosed with AxSpA who presented for consultation at the outpatient clinic. The diagnosis was established according to either the New York Criteria or the ASAS international working group (Assessment of SpondyloArthritis International Society) [3, 4]. Eligible patients regularly attended the outpatient Spondyloarthritis Clinic at the Hospital de Clínicas de Porto Alegre (HCPA). The HCPA is a tertiary medical center affiliated with the Medical School of the Universidade Federal do Rio Grande do Sul (UFRGS). It primarily handles and treats more severe cases, standing as a key referral institution for intricate medical conditions in the region. All subjects willingly signed an Informed Consent Form (ICF), which had been approved by the HCPA’s ethics committee (Certificado de Apresentação de Apreciação Ética – CAAE – number 01524112.2.0000.5327).

### Clinical and laboratory variables

Collected clinical and epidemiological data comprised age, gender, skin color, disease duration, and presence of psoriasis, uveitis, enthesitis, peripheral arthritis, dactylitis, and sacroiliitis, as confirmed by conventional radiology. HLA-B27 typing was performed for all participants using PCR-SSP (PCR—Sequence Specific Primer) and FC. Data were recorded in the Research Electronic Data Capture (REDCap) database, provided by the HCPA Research and Postgraduate Group.

The FC analyses were conducted in an external support laboratory, while the PCR-SSP tests were performed at HCPA with reagents developed by HCPA. For both tests, a volume of 5.0 mL of venous blood was drawn into a sterilized test tube containing 5.0% Ethylenediamine tetraacetic acid (EDTA) as an anticoagulant. The samples were stored in a refrigerator and processed within 24 h from the time of collection.

### Analytical methods employed

#### Flow cytometry (CF)

**Fig. 1:**
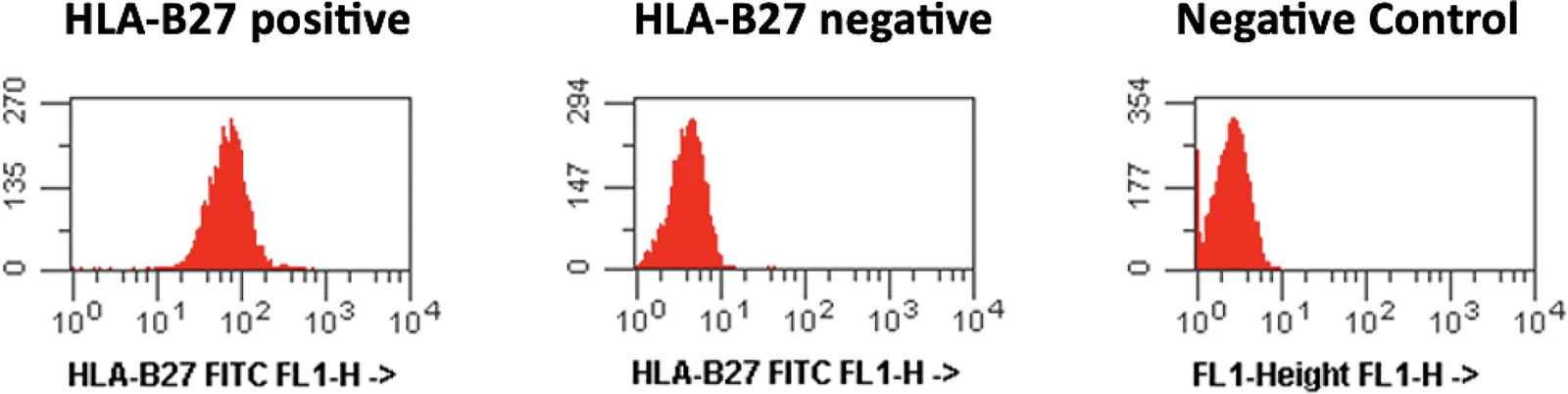
Marker expression graphs obtained using Flow Cytometry. The peak that overlaps with the negative control indicates a negative expression of the marker. In contrast, peaks to the right of the negative control peak denote positive marker expression. This image was provided as a courtesy of the support laboratory where the analyses were conducted

Whole blood analyses via FC followed the protocol described by Arlindo et al. [23], with some modifications based on recent literature. Briefly, 50 µL of EDTA-anticoagulated peripheral blood was incubated with 10 µL of CYTOGNOS® reagent containing two conjugated monoclonal antibodies: FITC-labeled HLA-B27 and PE-labeled HLA-B7 (CYT-COMB1012, Cytognos, SL, ESP). After 20 mins of dark incubation at room temperature, erythrocytes underwent lysis with Excellyse I® (EXBIO, Praha, CZ) for 5 mins, followed by distilled water for 10 mins. Samples were centrifuged and re-suspended in phosphate-buffered saline. Immediate data acquisition was done post-quality control procedures on a 4-color FACSCalibur® flow cytometer (BD Biosciences, San Diego, CA, USA), using CellQuestTM Pro software. Approximately 100,000 events were acquired per sample. CytoPaintClassic 1.1 software (Leukobyte, Pleasanton, CA, USA) was used for graphic analysis. Post debris exclusion, the lymphocyte region was highlighted, examining HLA-B27 and HLA-B7 expression. Positive and negative events were determined using an unmarked cell negative control. Data interpretation drew from graphical information (Fig. 1).

The inclusion of HLA-B7, alongside HLA-B27, was strategic to mitigate potential cross-reactivity issues inherent to these closely related alleles, which would lead to an increased false positive rate. This decision was informed by the known cross-reactive group (CREG) relationships, particularly between HLA-B27 and HLA-B7, which have been documented to share epitopes leading to cross-reactivity in some testing scenarios [24]. By having a positive HLA-B27 at high intensity without detecting HLA-B7, we can ascertain with high confidence that the result is indeed due to positive HLA-B27. Conversely, if both HLA-B27 and HLA-B7 are positive, the result may be considered indeterminate due to their cross-reactivity. The preference for using HLA-B7 instead of multiple monoclonal antibodies was due to feasibility and also because many available clones also share this similar problem of cross-reactivity [25].

#### Polymerase chain reaction (PCR)

The HLA typing method employed was the Polymerase Chain Reaction with Sequence-Specific Priming (PCR-SSP), following an in-house protocol, which served as the reference for this study.

DNA was extracted from each of the samples through the hemolysis of 5.0 mL of whole blood with EDTA, using the salting-out technique. Primers designed to specifically amplify a 127 bp fragment of the HLA-B27 gene from exon 2 were used. The forward primer sequence was 5′-GCTACGTGGACGACACGCT-3′, and reverse primers were 5′-CTCGGTCA GTCTGTGCCTT-3′ and 5′-TCTCGGTAAGTCTGTGCCTT-3′, targeting positions 76–94 and 207–226 of exon 2, respectively. This primer set was chosen for its ability to encompass alleles HLA-B27*01, B*27:02, B*27:03, B*27:04, and B*27:05.

For internal control of the reaction, primers targeting HLA-DRB1 intron 3 were used to amplify a 796 bp fragment. The sequences for these primers were 5′-TGCCAAGTG GAGCACCCAA-3′ and 5′-GCATCTTGCTCTGTGCAGAT-3′. This internal control ensures the amplification process’s efficacy for each sample, providing a reliability measure for the PCR reaction.

The PCR reaction mixture consisted of 1.0 µL of the obtained DNA, 1.5 µL of primers, and 7.0 µL of a master mix (50 µL of TDMH (Tris, dNTPs, MgCl2, and H2O), 24.5 µL of ultrapure water, and 1.0 µL of Taq Polymerase). This mixture was subjected to 31 cycles in a thermocycler (Applied Biosystems®), under the following conditions: cycle I: 96 °C for 60 s (once); cycle II: 96 °C for 20 s, 70 °C for 45 s, and 72 °C for 25 s (repeated five times); cycle III: 96 °C for 25 s, 65 °C for 50 s, and 72 °C for 30 s (repeated twenty-one times); cycle IV: 96 °C for 30 s, 55 °C for 60 s, and 72 °C for 90 s (repeated five times); and cycle V: 20 °C for 60 s (once). After completion, the samples were maintained at 4 °C indefinitely.

**Fig. 2:**
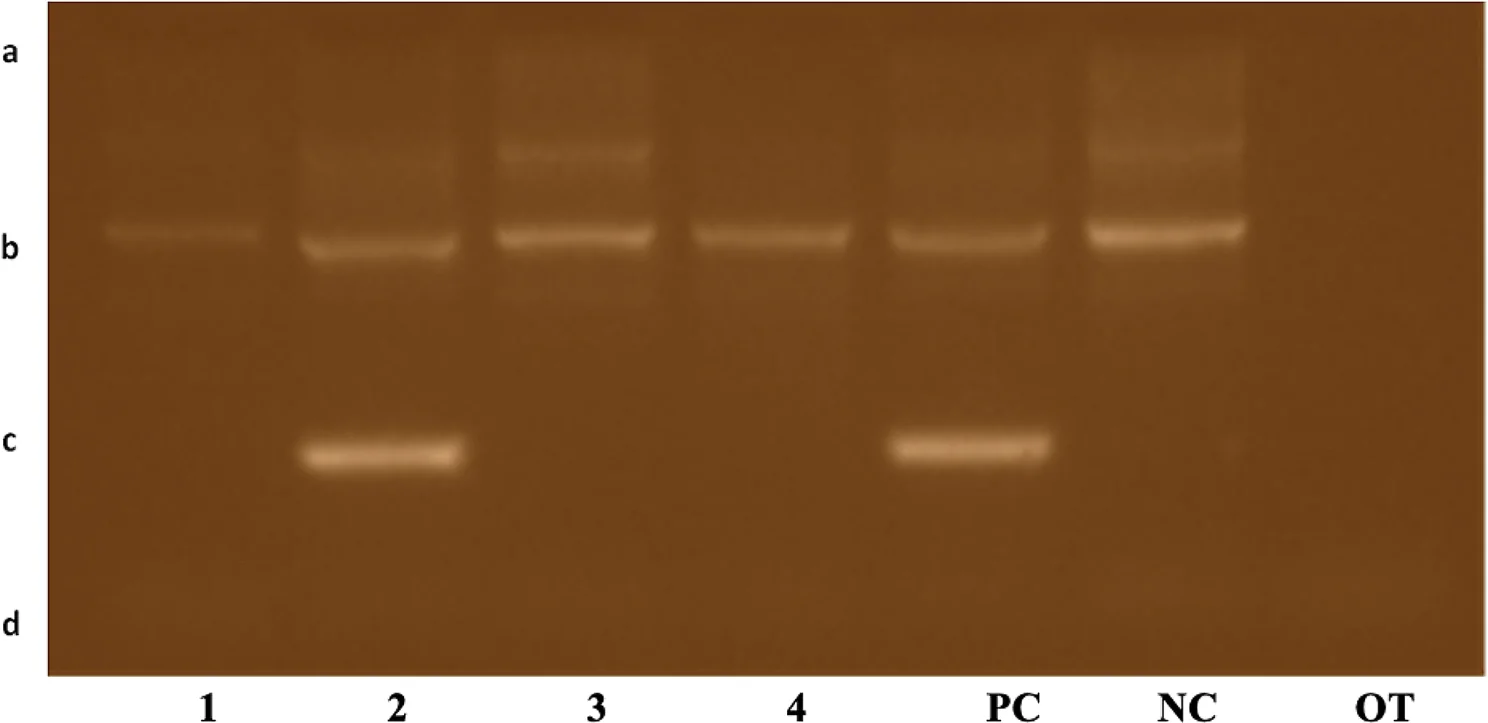
Ethidium bromide-stained agarose gel following electrophoretic run. Results of HLA-B27 genotyping by in-house PCR-SSP with an amplified fragment of 127 base pairs. Lanes 1–4: patients; *NC* negative control, *PC* positive control, *OT* open tube (beta-hemoglobin); **a** well; **b** internal control band (796 bp); **c** positive typing band (150 bp); **d** primer band

Gel electrophoresis was performed by mixing twenty-two microliters of the PCR-amplified material in the with 7.0 µL of an Orange G dye for visualization and loading onto a 2.0% agarose gel containing ethidium bromide. The electrophoresis was conducted at a voltage of 75 V and 170 mA for 30 mins, followed by visualization under UV transluminator. The results were documented based on the presence of the internal control band (comprising 796 base pairs) and the HLA-B27 specific band (127 base pairs), as shown in Fig. 2. Additionally, every test included an internal control, a positive B27 control sample, and a negative B27 control sample. These control samples were genotyped by Polymerase Chain Reaction with Sequence-Specific Oligonucleotide probes (PCR-SSO) or Next-Generation Sequencing (NGS) to ensure the assay’s accuracy and specificity.

### Statistical analysis

Data entry was performed using Microsoft Excel and was subsequently imported to SPSS v. 20.0 for statistical analysis. Categorical variables were summarized by frequencies and percentages. Quantitative variables with a symmetrical distribution were described by their mean and standard deviation. In contrast, those with an asymmetrical distribution were presented using the median and interquartile range.

The Fisher’s Exact Test was employed to compare categorical variables. For quantitative variables with symmetrical distribution, the Student’s *t*-test for independent samples was utilized. Variables with asymmetrical distribution were compared using the Mann-Whitney U test.

Performance measures for FC, considering PCR as the gold standard, were calculated. True positives (TP) were defined as patients who tested positive for HLA-B27 by both PCR and FC, while true negatives (TN) were those negative by both methods. False positives (FP) were cases positive by FC but negative by PCR, and false negatives (FN) were those negative by FC but positive by PCR. These definitions allowed us to accurately calculate sensitivity, specificity, positive predictive value (PPV), negative predictive value (NPV), and overall accuracy of the FC method. Additionally, the concordance between FC and PCR was evaluated using the Kappa statistic, with a significance level of 5% set for all statistical analyses.

## Results

The study enrolled 62 patients diagnosed with AxSpA, of whom 60 had r-AxSpA and 2 had nr-AxSpA. Among these, 40 (64.5%) were male, and 55 (88.7%) self-identified as Caucasian. The average age was 54.5 ± 12 years. The median disease duration since diagnosis, as indicated by the 25th and 75th percentiles, was 14 years, ranging between 9 and 24 years. There was a numerical difference when comparing the median disease duration between groups, with longer duration in the HLA-B27 negative group (*p* = 0.069).

The observed frequencies for the studied clinical features were as follows: psoriasis (4.8%), uveitis (40.3%), enthesitis (45.2%), peripheral arthritis (27.5%), dactylitis (14.5%), and sacroiliitis (96.8%). Table 1 provides a detailed breakdown of these findings and assesses the performance variance of clinical characteristics in relation to the presence or absence of the HLA-B27 antigen as detected by PCR. A statistically significant difference was observed for peripheral arthritis, with a higher frequency in the HLA-B27 negative group compared to the HLA-B27 positive group (*p* = 0.032).

**Table 1:** Demographic, clinical, and laboratory characteristics of the studied population according to the presence of the HLA-B27 antigen detected by PCR

Characteristics	*N* = 62	HLA-B27 status		*p* value
		Positive (*n* = 48)	Negative (*n* = 13)	
Male gender (%)	40 (64.5)	30 (62.5)	10 (76.9)	0.348
Caucasian (%)	55 (88.7)	45 (91.8)	10 (76.9)	0.153
Mean age (SD)	54.5 (12.3)	53.2 (12.5)	59.5 (10.5)	0.702
Median disease duration (IQR)	14 (9; 24)	13 (9; 21)	23 (11; 35)	0.069
Psoriasis (%)	3 (4.8)	2 (4.1)	1 (7.7)	0.513
Uveitis (%)	25 (40.3)	18 (36.7)	7 (53.8)	0.344
Enthesitis (%)	28 (45.2)	22 (44.9)	6 (46.2)	0.999
Peripheral arthritis (%)	17 (27.4)	10 (20.4)	7 (53.8)	0.032
Dactylitis (%)	9 (14.5)	7 (14.3)	2 (15.4)	0.999
Radiographic sacroiliitis (%)	60 (96.8)	47 (95.9)	13 (100.0)	0.999

*N*, number, *SD* standard deviation, *IQR* interquartile range

The correlation analysis results between FC and PCR, with PCR serving as the gold standard for comparison to FC, are presented in Table 2. Of the 62 patients examined, 56 (90.3%) were found positive for HLA-B27 typing using FC, while 49 (79%) were positive when tested via the PCR method. Notably, three of these FC-positive patients (5.3%) yielded indeterminate results when evaluated solely by FC, but were confirmed positive by PCR. The sensitivity of FC, which measures the proportion of true positives correctly identified by the test (i.e., also positive to the PCR test), was determined to be 98%. Regarding specificity, five patients (8.1%) were correctly identified as negative by both FC and PCR testing (true negatives), while eight patients (12.9%) were incorrectly identified as positive by FC but were negative by PCR (false positive). This established a specificity of 38.5% for FC. Among all the analyses, 53 (85.5%) showed consistent findings with both methods, but nine (14.5%) had discrepant results. The PPV of FC was calculated to be 85.7%, and its NPV was 83.5%. Consequently, the overall accuracy of FC was ascertained to be 85.5%. The Kappa statistic, employed to assess the concordance between the two testing methods, yielded a moderate agreement (kappa value of 0.454).

**Table 2:** Comparison of the HLA-B27 results using PCR and FC techniques

		Polymerase chain reaction		Total
		Negative	Positive	
Flow Cytometry	Negative	5 (8.1%)	1 (1.6%)	6 (9.7%)
	Positive	8 (12.9%)	48 (77.4%)	56 (90.3%)
Total cases		13 (21%)	49 (79%)	62 (100%)

## Discussion

The pivotal role of HLA-B27 in the diagnosis and prognosis of AxSpA underscores the importance of precise detection methodologies. Traditional diagnostic techniques, such as FC, have been widely utilized due to their cost-effectiveness, straightforward implementation, and high sensitivity. However, its specificity in detecting HLA-B27 has been debated, prompting consideration of PCR-based methods, such as PCR-SSP and PCR-SSO. These techniques, recognized for their ability to provide, simultaneously, high sensitivity and specificity, are seen as offering enhanced test accuracy for HLA-B27 testing [21, 26].

Despite the acknowledged specificity benefits of PCR-SSP and PCR-SSO, along with the ASAS designating HLA-B27 as a critical clinical criterion for AxSpA, there remains a lack of consensus on a universally recommended testing method [4]. This absence of a definitive endorsement likely mirrors the array of diagnostic practices across various clinical settings. Moreover, the observation by the College of American Pathologists (CAP) that FC remains the dominant method for HLA-B27 testing in the United States, accounting for 52% of all tests in 2020 [22], highlights the nuanced decision-making process imposed by the need to balance diagnostic precision against considerations of accessibility and cost.

In alignment with prior research, our study has demonstrated that FC exhibits notable sensitivity in detecting HLA-B27. Our observed sensitivity not only surpassed that reported by Del Muñoz-Villanueva et al. in 2000 [27] but also proved comparable to the findings of Lingerfelter et al. in 1995 [28]. Upon further examination, it becomes evident that disparities in sensitivity across these and other studies can be attributed to a combination of factors. While sample quality is undeniably critical for FC, given its reliance on ample, viable cell-containing material, the choice of monoclonal antibodies emerges as a paramount determinant of assay performance. The importance of proper sample storage was illustrated by Seo et al., in 2013, where they compared four different storage methods and found ideal results when using frozen platelets (sensitivity of 100% and specificity of 99.3%) [19]. Moreover, the choice of monoclonal antibodies necessitates careful consideration to minimize the risk of cross-reactivity [29], a prevalent challenge in FC that can lead to false-positive results. Selecting antibodies with low cross-reactivity potential is crucial in enhancing the assay’s diagnostic precision, underlining the relationship between antibody selection and the overall reliability of FC in HLA-B27 testing.

While FC exhibits high sensitivity in detecting HLA-B27, akin to its role in other serological assessments, it shares certain limitations that can affect diagnostic precision due to lack of specificity. Owing to cross-reactivity with certain HLA alleles, these assays occasionally require confirmation by more precise techniques. In our cohort of 62 patients, 90.3% tested positive for HLA-B27 via FC, compared to 79% identified by the PCR method. Although FC’s sensitivity stood at 98%, the specificity was observed to be lower (38.5%), indicating a potential for ruling out non-HLA-B27-associated conditions. This discrepancy was evident in the alignment of results between FC and PCR in 95.5% of cases, with a discordance rate of 14.5%. Such variability, alongside a Kappa statistic of 0.45, highlights the need for enhanced specificity in FC testing, particularly due to potential cross-reactivity, notably with the HLA-B7 allele [19]. To mitigate these specificity challenges, it is advocated to use a minimum of two different anti-HLA-B27 monoclonal antibodies in the FC assay [30, 31]. This approach aims to refine diagnostic accuracy by reducing false-positive outcomes attributed to allele cross-reactivity.

While we have highlighted the high sensitivity of FC in detecting HLA-B27, it is imperative to also consider its limitations, especially when compared to molecular testing methods. FC, despite its cost-effectiveness and rapid turnaround time, may not achieve the same level of accuracy as molecular tests. Specifically, PCR-SSO and NGS methods have emerged as more refined approaches for HLA typing, providing allele-level resolution that FC cannot match [26]. Another significant limitation of FC in the context of HLA-B27 testing is its inability to discriminate between alleles associated with AxSpA and those that are not, such as B27*06 or B27*09 [21]. This lack of specificity can lead to false-positive results, as FC does not differentiate between the various subtypes of the HLA-B27 allele. Despite these challenges, FC remains a valuable tool for HLA-B27 screening, particularly in resource-limited settings.

Our analysis, therefore, reflects a broader diagnostic dilemma: the balance between FC’s sensitivity and the need for greater specificity as provided by PCR. Recently, the notion of value-based healthcare (VBHC) in rheumatology has gained momentum due to the rising medical expenses, which often limit access to ideal care in many regions of the globe [32, 33]. Essentially, VBHC prioritizes patient-centric outcomes while curtailing unnecessary expenses, like judicious use of HLA-B27 testing in suspected AxSpA cases [34]. The American College of Rheumatology (ACR) and the Canadian Rheumatology Association (CRA) recently published their Choosing Wisely recommendations (CWRs). Both bodies emphasized the judicious selection of HLA-B27 tests to eschew needless expenses and avert misdiagnoses. Specifically, they advocate for testing in the presence of two or more features suggestive of AxSpA, or one feature coupled with radiographic sacroiliitis [35, 36].

**Fig. 3:**
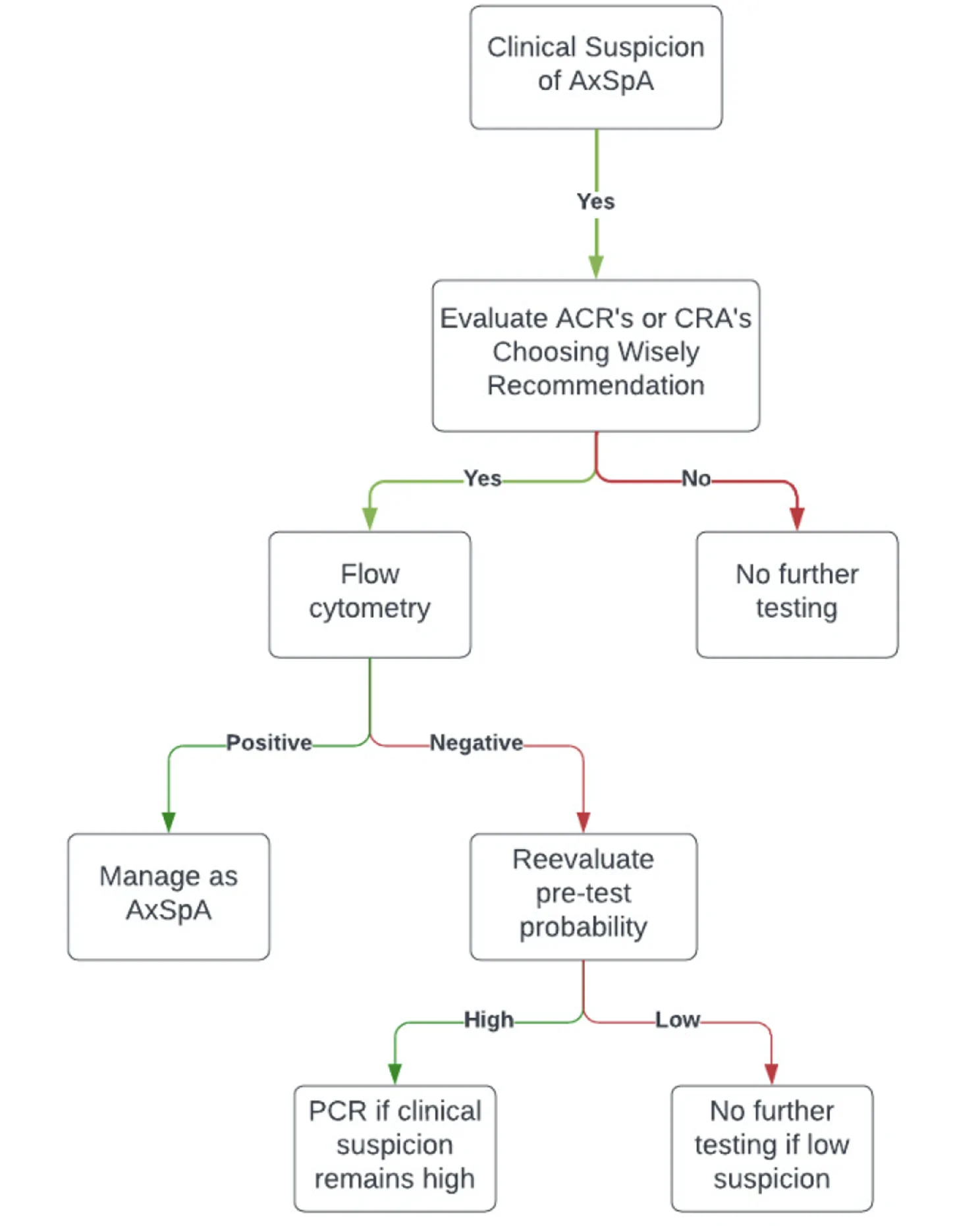
Diagnostic algorithm. Initially, upon a clinical suspicion of AxSpA, determine if the clinical scenario meets the ACR’s or CRA’s Choosing Wisely Recommendations (CWRs). If so, proceed with Flow Cytometry (FC) as the primary diagnostic test. Patients with positive FC results are managed as AxSpA. In contrast, if the FC result is negative and the clinical suspicion remains high, further testing with PCR is advised. For those not meeting the CWRs criteria or having negative findings without continued clinical suspicion, no further testing is recommended, effectively optimizing both diagnostic accuracy and cost-efficiency. *AxSpA* axial spondyloarthritis, *ACR* American College of Rheumatology, *CRA* Canadian Rheumatology Association, *PCR* polymerase chain reaction

Yet, the gap between recommendation and actual practice is stark. A recent retrospective study conducted in Minnesota highlighted this disparity: merely 35% of rheumatologists complied with these guidelines, and a mere 13.9% of primary care practitioners did the same [37]. Therefore, to emphasize a VBHC in the field of rheumatology, especially in lower-income regions, we propose an algorithm that acknowledges the strengths of each test and the CWRs (Fig. 3). For instance, at HCPA, an average of 88 HLA-B27 tests are ordered annually. Currently, PCR is priced at $64.00, whereas FC costs roughly $18.00. By following the CWRs, only 35% of the tests, or 31 out of 88 tests, would be ordered by rheumatologists. This refinement in test ordering would translate to direct savings of $3648.00. Employing FC as the primary diagnostic tool for these 31 patients results in a total expense of $558.00—significantly less than the $1984.00 if all underwent PCR testing. Notably, considering the sensitivity of FC at 98%, there is likely to be 1 false negative case, which could require retesting via PCR. Additionally, our data suggests that 5.3% of the FC tests might yield indeterminate results (i.e., 2 patients), necessitating a follow-up PCR test. These scenarios necessitate follow-up PCR tests for a total of 3 patients, costing an additional $192.00. By implementing this algorithm, the annual diagnostic expenditure at HCPA would be $750.00, a stark contrast to the potential $5632.00, demonstrating a considerable cost-saving at each diagnostic phase. This methodology not only optimizes expenditures but also guarantees patients receive prompt and effective diagnostic care. (All financial figures are in US dollars, based on the dollar-real exchange rate as of August 14th, 2023.)

HLA-B27 prevalence was 79% in our study, which aligns with findings by Sampaio-Barros et al. in 2013. Moreover, we observed that 91.8% of HLA-B27 seropositive patients self-identified as Caucasian, consistent with the earlier review of the Brazilian consensus on spondyloarthropathies from 2007 [9]. Among the clinical characteristics of our study population, the presence of peripheral disease combined with a smaller proportion of HLA-B27 positive patients underscores the gene’s association with axial involvement. This demographic and clinical insight further emphasizes the need for understanding the specificities of the patient population when interpreting diagnostic outcomes.

Accurate detection of HLA-B27 supports clinicians in disease diagnosis, and the chosen method should take into account factors such as sensitivity, specificity, test availability, and cost implications for both the patient and the health system, be it public or private. With the increasing accessibility of knowledge and information, the integration of molecular biology into lab routines is advancing. Nevertheless, its adoption remains elusive for many smaller facilities in countries like Brazil, the US and India [22, 38]. Techniques like PCR require standardized facilities, trained staff, and specific equipment to ensure their quality execution. These requirements hinder many establishments from offering these tests. Efforts to address these challenges invariably increase costs, further elevating test expenses [18]. Conversely, implementing a flow cytometer does not require regulated space; equipment handling follows the manufacturer’s guidelines, and the technique is based on the reagent kit’s instructions. Despite its limitations, novel devices are emerging to bridge these gaps, aiming to simplify sample handling or to enhance the equipment’s sensitivity and specificity by augmenting the number of fluorochromes and lasers [39, 40].

Several limitations need to be acknowledged in our study. Firstly, our patient selection relied on convenience sampling, potentially introducing selection bias and questioning the generalizability of our findings to the broader axSpA patient population. A notable observation was that the majority of participants satisfied the New York criteria for sacroiliitis. While this might hint at a selection bias, skewing our cohort towards a high pre-test likelihood, it could also have ramifications on the perceived accuracy of the tests. The outcomes might seem more decisive than they would in a heterogeneous or more ambiguous patient group. Yet, this very characteristic could be interpreted as a strength: diagnostic accuracy assessments are most meaningful in populations without diagnostic ambiguity. Additionally, while the molecular methods used were of the latest design, they were restricted to reagents exclusively developed by HCPA, which might affect the reproducibility of the results in other settings or using alternative reagents. Lastly, while our sample size was robust, larger multi-center studies may offer more comprehensive insights and validation of our findings in diverse populations and geographical areas. Future studies could benefit from a prospective design, randomized sampling, and inclusion of the latest diagnostic markers to enhance the rigor and external validity of the findings.

In summary, while the stakes in medical diagnosis are invariably high, ensuring accurate and cost-effective diagnostic procedures is pivotal. Given its relative affordability, simpler handling, and quicker result delivery than PCR, FC might still serve at least as a preliminary screening tool in certain scenarios. It can assist physicians in ruling out false negatives, thereby streamlining their investigation for AxSpA diagnosis while decreasing costs, which is paramount in the current landscape of steeping medical costs. Our proposed algorithm presents a possible framework for healthcare systems striving to balance diagnostic accuracy with financial prudence. Future investigations exploring the root causes behind test discrepancies—whether inherent to the tests, patient-specific, or due to technical nuances—can propel the field towards more reliable, consistent, and patient-centric diagnostic modalities.

## Data Availability

All data generated or analyzed during this study are included in this published article.

## References

[1] Taurog JD, Chhabra A, Colbert RA. Ankylosing spondylitis and Axial Spondyloarthritis. N Engl J Med. 2016;374(26):2563–74. 27355535 10.1056/NEJMra1406182

[2] López-Medina C, Molto A, Sieper J, Duruöz T, Kiltz U, Elzorkany B, et al. Prevalence and distribution of peripheral musculoskeletal manifestations in spondyloarthritis including psoriatic arthritis: results of the worldwide, cross-sectional ASAS-PerSpA study. RMD Open. 2021;7(1):e001450–001450. 33462157 10.1136/rmdopen-2020-001450 PMC7816910

[3] Moll JM, Wright V. New York clinical criteria for ankylosing spondylitis. A statistical evaluation. Ann Rheum Dis. 1973;32(4):354–63. 4269429 10.1136/ard.32.4.354 PMC1006116

[4] Rudwaleit M, van der Heijde D, Landewé R, Listing J, Akkoc N, Brandt J, et al. The development of Assessment of SpondyloArthritis international society classification criteria for axial spondyloarthritis (part II): validation and final selection. Ann Rheum Dis. 2009;68(6):777–83. 19297344 10.1136/ard.2009.108233

[5] Ramiro S, Nikiphorou E, Sepriano A, Ortolan A, Webers C, Baraliakos X, et al. ASAS-EULAR recommendations for the management of axial spondyloarthritis: 2022 update. Ann Rheum Dis. 2023;82(1):19–34. 36270658 10.1136/ard-2022-223296

[6] Capelusnik D, Zhao SS, Boonen A, Ziade N, Medina CL, Dougados M, et al. Individual-level and country-level socio-economic factors and health outcomes in spondyloarthritis: analysis of the ASAS-perSpA study. Rheumatol Oxf Engl. 2022;61(5):2043–53. 10.1093/rheumatology/keab638 34387300

[7] Citera G, Bautista-Molano W, Peláez-Ballestas I, Azevedo VF, Perich RA, Méndez-Rodríguez JA et al. Prevalence, demographics, and clinical characteristics of latin American patients with spondyloarthritis. Adv Rheumatol. 2021;61(1). 10.1186/s42358-020-00161-5 33419481

[8] Feldtkeller E, Khan MA, van der Heijde D, van der Linden S, Braun J. Age at disease onset and diagnosis delay in HLA-B27 negative vs. positive patients with ankylosing spondylitis. Rheumatol Int. 2003;23(2):61–6. 12634937 10.1007/s00296-002-0237-4

[9] Sampaio-Barros PD, Azevedo VF, Bonfiglioli R, Campos WR, Carneiro SC da S, Carvalho MAP, et al. Consenso Brasileiro de Espondiloartropatias: espondilite anquilosante e artrite psoriásica diagnóstico e tratamento - primeira revisão. Rev Bras Reumatol. 2007;47:233–42.

[10] Sieper J, Braun J, Dougados M, Baeten D. Axial spondyloarthritis. Nat Rev Dis Primer. 2015;1:15013. 10.1038/nrdp.2015.13 27188328

[11] Kavadichanda CG, Geng J, Bulusu SN, Negi VS, Raghavan M. Spondyloarthritis and the Human Leukocyte Antigen (HLA)-B*27 connection. Front Immunol. 2021;12:601518. 33763060 10.3389/fimmu.2021.601518 PMC7982681

[12] González S, Martínez-Borra J, López-Larrea C, Immunogenetics. HLA-B27 and spondyloarthropathies. Curr Opin Rheumatol. 1999;11(4):257–64. 10411379 10.1097/00002281-199907000-00006

[13] Brewerton DA, Hart FD, Nicholls A, Caffrey M, James DC, Sturrock RD. Ankylosing spondylitis and HL-A 27. Lancet Lond Engl. 1973;1(7809):904–7. 10.1016/s0140-6736(73)91360-3 4123836

[14] Sampaio-Barros PD. Epidemiology of spondyloarthritis in Brazil. Am J Med Sci. 2011;341(4):287–8. 21358306 10.1097/MAJ.0b013e31820f8caf

[15] Resende GG, Saad CGS, de Oliveira DCM, de Sousa Bueno Filho JS, Sampaio-Barros PD, de Medeiros Pinheiro M. HLA-B27 positivity in a large miscegenated population of 5,389,143 healthy blood marrow donors in Brazil. Adv Rheumatol. 2023;63(1):16. 37081582 10.1186/s42358-023-00302-6

[16] Freeston J, Barkham N, Hensor E, Emery P, Fraser A. Ankylosing spondylitis, HLA-B27 positivity and the need for biologic therapies. Joint Bone Spine. 2007;74(2):140–3. 17350312 10.1016/j.jbspin.2006.11.003

[17] Resende GG, Meirelles E de S, Marques CDL, Chiereghin A, Lyrio AM, Ximenes AC, et al. The Brazilian society of Rheumatology guidelines for axial spondyloarthritis– 2019. Adv Rheumatol. 2020;60(1):19. 32171329 10.1186/s42358-020-0116-2

[18] Cho EH, Lee SG, Seok JH, Park BYN, Lee EH. Evaluation of two commercial HLA-B27 real-time PCR kits. Korean J Lab Med. 2009;29(6):589–93. 20046093 10.3343/kjlm.2009.29.6.589

[19] Seo BY, Won DI. Flow cytometric human leukocyte antigen-B27 typing with stored samples for batch testing. Ann Lab Med. 2013;33(3):174–83. 23667843 10.3343/alm.2013.33.3.174 PMC3646191

[20] Seipp MT, Erali M, Wies RL, Wittwer C. HLA-B27 typing: evaluation of an allele-specific PCR melting assay and two flow cytometric antigen assays. Cytometry B Clin Cytom. 2005;63(1):10–5. 15624199 10.1002/cyto.b.20039

[21] Chheda P, Warghade S, Mathias J, Dama T, Matkar S, Shah N, et al. HLA-B27 testing: a journey from flow cytometry to molecular subtyping. J Clin Lab Anal. 2018;32(5):e22382. 29349813 10.1002/jcla.22382 PMC6817030

[22] Peña JRA, Fung MK, Gandhi MJ. A review of Laboratory practices using the HLA-B27 survey by the College of American pathologists: how important is allele-level typing? Arch Pathol Lab Med. 2023. 10.5858/arpa.2022-0322-CP 37134231

[23] Arlindo EM, Marcondes NA, Fernandes FB, Faulhaber GAM. Quantitative flow cytometric evaluation of CD200, CD123, CD43 and CD52 as a tool for the differential diagnosis of mature B-cell neoplasms. Rev Bras Hematol E Hemoter. 2017;39(3):252–8. 10.1016/j.bjhh.2017.05.002 PMC5567423 28830605

[24] Wade JA, Katovich Hurley C, Takemoto SK, Thompson J, Davies SM, Fuller TC, et al. HLA mismatching within or outside of cross-reactive groups (CREGs) is associated with similar outcomes after unrelated hematopoietic stem cell transplantation. Blood. 2007;109(9):4064–70. 17202313 10.1182/blood-2006-06-032193 PMC1874562

[25] Waeckel L, Kennel A, Berger AE, Lambert C. Human leukocyte antigen-B27 typing by flow cytometry: comparison of three CE-IVD methods. Cytometry B Clin Cytom. 2023;104(6):460–7. 36448700 10.1002/cyto.b.22102

[26] Profaizer T, Dibb K, Bethers H, Monds C, Andreasen J, Delgado JC, et al. Comparison of Next-Generation sequencing-based Human Leukocyte Antigen typing with clinical Flow Cytometry and Allele-specific PCR melting assays for HLA-B27 genotyping. J Appl Lab Med. 2021;6(5):1221–7. 34151972 10.1093/jalm/jfab046

[27] Muñoz-Villanueva M, Muñoz E, Veroz R, Escudero Contreras A, Torre MJ, Gonzalez R, et al. Differences in the recognition of HLA-B27 by microlymphocytotoxicity and flow cytometry. Determination of B27 subtypes in andalusian patients with spondiloarthropathies. Rev Esp Reumatol. 2000;27:120–7.

[28] Lingenfelter B, Fuller TC, Hartung L, Hunter J, Wittwer C. HLA-B27 screening by flow cytometry. Cytometry. 1995;22(2):146–9. 7587746 10.1002/cyto.990220211

[29] Levering WHBM, Wind H, Sintnicolaas K, Hooijkaas H, Gratama JW. Flow cytometric HLA-B27 screening: cross-reactivity patterns of commercially available anti-HLA-B27 monoclonal antibodies with other HLA-B antigens. Cytometry B Clin Cytom. 2003;54(1):28–38. 12827665 10.1002/cyto.b.10022

[30] Bonnaud G, Aupetit C, Preux PM, Cogné M, Drouet M. Optimisation of HLA-B27 testing by association of flow cytometry and DNA typing. Clin Rheumatol. 1999;18(1):23–7. 10088944 10.1007/s100670050046

[31] Baráth S, Mezei ZA, Száraz-Széles M, Hevessy Z. Combined use of different antibody clones improves the efficiency of human leukocyte antigen B27 detection by flow cytometry. Cytometry B Clin Cytom. 2022;102(3):239–45. 33373171 10.1002/cyto.b.21989

[32] Ribeiro AL, Dullius L, Sartori NS, Azeredo-da-Silva A, Kohem CL, Coates L, et al. Challenges in the management of psoriatic arthritis in Latin America: a systematic review. Clin Ther. 2023;S0149–2918(23):00134–0. 10.1016/j.clinthera.2023.04.005 37198042

[33] Pineda C, Sandoval H, Sheen R, Muñoz-Louis RPANLAR, Presidency FS-AR. 2014–2016: challenges, opportunities, and results. J Clin Rheumatol Pract Rep Rheum Musculoskelet Dis. 2017;23(2):107–12. 10.1097/RHU.0000000000000515 28225512

[34] Liew DFL, Dau J, Robinson PC. Value-based Healthcare in Rheumatology: Axial Spondyloarthritis and Beyond. Curr Rheumatol Rep. 2021;23(6):36. 33909169 10.1007/s11926-021-01003-z

[35] Chow SL, Carter Thorne J, Bell MJ, Ferrari R, Bagheri Z, Boyd T, et al. Choosing wisely: the Canadian Rheumatology Association’s list of 5 items physicians and patients should question. J Rheumatol. 2015;42(4):682–9. 25641889 10.3899/jrheum.141140

[36] Yazdany J, Schmajuk G, Robbins M, Daikh D, Beall A, Yelin E, et al. Choosing wisely: the American College of Rheumatology’s top 5 list of things physicians and patients should question. Arthritis Care Res. 2013;65(3):329–39. 10.1002/acr.21930 PMC4106486 23436818

[37] Choosing Unwisely. HLA-B27 Testing and Adherence to Choosing Wisely Practices in a Large Integrated Academic Healthcare System [Internet]. ACR Meeting Abstracts. [cited 2023 Aug 11]. https://acrabstracts.org/abstract/choosing-unwisely-hla-b27-testing-and-adherence-to-choosing-wisely-practices-in-a-large-integrated-academic-healthcare-system/.

[38] Priyathersini N, Shanmugam SG, Devi SS, Chinambedu Dandapani MP, Rajendiran S, D’Cruze L. Detection of Human Leukocyte Antigen B27 by Flowcytometry in patients with suspected Ankylosing spondylitis in a Tertiary Care Centre. Cureus. 2021;13(2):e13560. 10.7759/cureus.13560 PMC8004549 33791178

[39] Aarsand AK, Johannessen HB, Scott CS. Evaluation of a method for monoclonal antibody HLA-B27 analysis with the CELL-DYN sapphire haematology analyser. Int J Lab Hematol. 2007;29(6):454–60. 17988301 10.1111/j.1751-553X.2007.00912.x

[40] Eissing N, Heger L, Baranska A, Cesnjevar R, Büttner-Herold M, Söder S, et al. Easy performance of 6-color confocal immunofluorescence with 4-laser line microscopes. Immunol Lett. 2014;161(1):1–5. 24726673 10.1016/j.imlet.2014.04.003

